# Latino Grandparents Raising Grandchildren: Cultural Assets and Support Strategies

**DOI:** 10.1093/geroni/igaf122.1172

**Published:** 2025-12-31

**Authors:** Nancy Mendoza

**Affiliations:** The Ohio State University, Columbus, Ohio, United States

## Abstract

The prevalence of Latino grandparents raising grandchildren continues to rise. In 2019, an estimated 1 out of 7 Latino children lived with a grandparent, and 1.6% were being raised solely by a grandparent, with no parent in the home. Despite the high prevalence of these grandfamilies, they remain under-researched and underserved. Limited research shows that cultural values such as familismo, machismo, and marianismo significantly impact their experiences. Effective supports should consider and incorporate Latino cultural values. In a climate where formal support services are becoming more limited and are often inappropriate for Latino grandparent caregivers, there is a greater reliance on and need for mutual aid. This is crucial as there is often reluctance to engage with formal service networks due to mistrust and fear of repercussions. By understanding how Latino culture influences the lived experiences of these grandparents, researchers and practitioners can become trusted allies. They can help Latino grandparent caregivers leverage their cultural assets to address the service and support gaps they may be experiencing. This presentation will begin with a summary of how Latino cultural practices such as multigenerational living, the grandparent role in helping to raise grandchildren, and a woman’s role in caring for the family can impact the experiences of Latino grandparent caregivers’ experiences both positively and negatively. It will conclude with suggestions on ways Latino cultural values can be incorporated as cultural assets for effective supportive strategies for Latino grandparents raising grandchildren.

